# Annual Meeting of the Earlville Union Medical Society

**Published:** 1859-01

**Authors:** D. C. Gould, D. M. Vosburgh

**Affiliations:** President; Secretary


### PROCEEDINGS OF MEDICAL SOCIETIES.

### ANNUAL MEETING OF THE EARLVILLE UNION MEDICAL SOCIETY.

The second annual meeting of this association was held at Earlville, July 13th, 1858, commencing at 2 o'clock p. m.

The meeting was called to order by the Vice-President, D. C. Gould, of Northville.

The minutes of the previous meeting were approved as recorded, and read by the Secretary.

An election of officers for the ensuing year was held, which resulted as follows;

| | | | |
|---|---|---|---|
| *President*, . . . | D. C. Gould, M.D., | of | Northville. |
| *Vice do.*, . . . | J. W. Edwards, | " " | Mendota. |
| *Secretary*, . . . | D. M. Vosburgh, | " " | Earlville. |
| *Treasurer*, . . . | S. Wiley, | " " | " |
| *Censors*, . . . . | P. E. Cook, | " " | Mendota. |
| | D. Hinckley, | " " | Leland. |
| | W. P. Sherwood, | " " | East Paw Paw. |

Interesting discussions were held amongst all the members present upon the following subjects: Bite of the Rattlesnake, Cholera, and Cholera Morbus.

The Secretary was instructed to furnish a copy of the proceedings to the *Chicago Medical Journal* for publication.

Then adjourned until the semi-annual meeting, which will be held on the 10th November next.

D. C. GOULD, *President*.

D. M. VOSBURGH, *Secretary*.

